# Characterization of a Virus Rescued from a Full-Length Infectious Clone Derived from the Type A Foot-and-Mouth Disease Virus Isolated in South Korea

**DOI:** 10.3390/v17121561

**Published:** 2025-11-29

**Authors:** Jae Young Kim, Sun Young Park, Gyeongmin Lee, Sang Hyun Park, Jong Sook Jin, Jong-Hyeon Park, Young-Joon Ko

**Affiliations:** Center for Foot-and-Mouth Disease Vaccine Research, Animal and Plant Quarantine Agency, 177 Hyeoksin 8-ro, Gimcheon-si 39660, Gyeongsangbuk-do, Republic of Korea; ivorikim@korea.kr (J.Y.K.); sun3730@korea.kr (S.Y.P.); lgm6004@korea.kr (G.L.); shpark0205@korea.kr (S.H.P.); in75724@korea.kr (J.S.J.); parkjhvet@korea.kr (J.-H.P.)

**Keywords:** foot-and-mouth disease, A/SKR/Yeoncheon/2017, infectious clone, vaccine, antigen productivity

## Abstract

Foot-and-mouth disease (FMD), a vesicular disease, causes lesions in the mouth, nose, teats, and feet of cloven-hoofed animals. Vaccination remains the most effective method to prevent FMD outbreaks. Since 2010, South Korea has implemented nationwide vaccination and developed multiple domestic vaccine strains to achieve vaccine self-sufficiency. Here, we aimed to construct an infectious clone using the A/SKR/Yeoncheon/2017 virus, which exhibits the highest antigen productivity among previously developed vaccine strains. An infectious clone was constructed based on the A/Yeoncheon/SKR/2017 virus isolated during an FMD outbreak in Korea in 2017. The viral genome was amplified in two fragments and assembled into a full-length clone, from which infectious recombinant virus was successfully rescued. The rescued virus was confirmed via serotyping and transmission electron microscopy to exhibit canonical 25–30 nm icosahedral morphology. Under optimized culture conditions using suspension-adapted BHK-21 cells (multiplicity of infection 0.001; 12 h post-infection), the recombinant virus achieved titers of 10^8^ TCID_50_/mL and produced 6.2 μg/mL of 146S antigen, comparable to its parental counterpart. The experimental vaccine formulated with the rescued virus (15 μg/dose), 1% saponin, 1% aluminum hydroxide gel, and ISA 206 VG, induced protective immunity in eight-week-old pigs, with vaccinated animals exhibiting no clinical signs following homologous challenge. To our knowledge, this study represents the first successful construction of an infectious clone derived from a field-isolated serotype A FMDV in South Korea. In the future, this A/SKR/Yeoncheon/2017 infectious clone can serve as a platform backbone for the rapid development of next-generation, high-yield vaccine seed strains through targeted epitope exchange.

## 1. Introduction

Foot-and-mouth disease (FMD) is a highly contagious transboundary viral disease that affects cloven-hoofed animals, including cattle, pigs, sheep, and goats. The disease is characterized by fever and vesicular lesions on the oral mucosa, feet, and teats, leading to substantial economic losses due to decreased productivity, trade restrictions, and the high cost of control measures. Because FMD virus (FMDV) is readily transmitted through aerosols, fomites, and animal movements, vaccination remains a central pillar of global FMD control strategies [1,2]. FMD is classified as a Category 1 legally notifiable livestock infectious disease under the Contagious Animal Disease Prevention Act in the Republic of Korea.

FMDV, the causative agent, is a non-enveloped virus containing a single-stranded positive-sense RNA genome. The capsid precursor P1 is cleaved to generate VP1–VP4, which assemble into protomers, pentamers, and ultimately the 146S intact virion. Moreover, its non-structural proteins (L, 2A, 2B, 2C, 3A, 3B, 3C, and 3D) are involved in viral replication in host cells [3,4,5,6].

Among the seven FMDV serotypes (O, A, C, Asia1, SAT1–3), serotype A exhibits the greatest genetic and antigenic heterogeneity, contributing to its complex epidemiology and demanding continuous adaptation of vaccine strains [7]. In Korea, FMD outbreaks have been reported since 2000, with additional cases recently recorded in 2025. Following the severe nationwide outbreaks in 2010–2011, vaccination has been implemented across all susceptible livestock [8]. Currently, all FMD vaccines used in Korea are imported, with efforts underway to establish domestic vaccine production facilities to ensure stable supply and vaccine sovereignty. Consequently, multiple vaccine strains and related production technologies have been developed domestically. The A/SKR/Yeoncheon/2017 virus isolated in Korea has been adapted for cell culture and previously developed as a vaccine strain to confer protective efficacy against various viral strains [9].

Traditional inactivated vaccines rely on field-isolated viruses that must undergo extensive cell-culture adaptation. However, such adaptation often results in unpredictable mutations that alter antigenicity or reduce antigen yield [10]. Infectious cDNA clones provide a powerful alternative, enabling rational design of vaccine backbones with optimized genetic stability and antigen productivity. Moreover, by substituting capsid-encoding regions with those from newly emerged field strains, infectious clones support rapid generation of vaccine seed viruses without prolonged in vitro adaptation [11,12]. Lee et al. reported the generation of seven FMDV serotypes based on a reverse genetics system by replacing P1 regions using the O1 Manisa backbone [11].

The A/SKR/Yeoncheon/2017 strain, isolated during an outbreak in South Korea, has been identified as a high-antigen-producing strain after successful adaptation to suspension culture [13]. Building on this advantage, we hypothesized that an infectious clone derived from this strain could serve as a versatile backbone for the development of next-generation, high-yield vaccine candidates. Here, we report the construction of a full-length infectious cDNA clone of the A/SKR/Yeoncheon/2017 virus and the successful rescue of infectious recombinant virus. We further compare its antigen productivity with that of the parental strain and evaluate its protective efficacy in pigs following homologous challenge. This study provides a foundational platform for accelerating the development of epitope-engineered FMD vaccines with enhanced antigen yield and rapid adaptability to evolving serotype A lineages.

## 2. Materials and Methods

### 2.1. Cells and Viruses

Fetal goat tongue cells (ZZ-R; Friedrich-Loeffler-Institute, Riems, Germany) were maintained under standard conditions in the Dulbecco’s modified Eagle’s medium/Ham’s F12 (Corning, Union City, NJ, USA). Adherent baby hamster kidney cells (BHK-21: C-13; ATCC CCL-10, Manassas, VA, USA) were propagated in the Dulbecco’s modified Eagle’s medium (Corning) supplemented with 10% fetal bovine serum (Gibco, Paisley, UK) and 1% antibiotic–antimycotic solution (Gibco) and incubated at 37 °C in a humidified atmosphere containing 5% CO_2_. A suspension-adapted BHK-21 cell line, co-developed by the Animal and Plant Quarantine Agency and Korea Research Institute of Bioscience and Biotechnology, was cultivated in the serum-free Cellvento BHK-200 medium (Merck, Darmstadt, Germany) at 37 °C with orbital shaking at 110 rpm. Cell density and viability were assessed via trypan blue exclusion using the Vi-Cell XR Automated Analyzer (Beckman Coulter, Brea, CA, USA). For viral inoculation, cultures containing 3 × 10^5^ cells/mL were grown for approximately 3.5 d to reach 3 × 10^6^ cells/mL. Subsequently, 30% fresh Cellvento medium was added to the culture, without removing the existing medium. The A/SKR/Yeoncheon/2017 strain of FMDV, originally isolated during South Korean outbreaks, was used to infect the suspension-adapted BHK-21 cells at 0.001 multiplicity of infection. The cultures were harvested 12 h post-infection (hpi), and supernatants were collected via centrifugation (4000× *g*, 20 min, 4 °C) for virus titration and quantification of 146S particles. Then, virus inactivation was performed with 3 mM binary ethylenimine (Sigma-Aldrich, St. Louis, MO, USA) at 26 °C for 28 h, followed by neutralization with 1 M sodium thiosulfate (Daejung Chemicals & Metals, Siheung, Republic of Korea) at a final concentration of 2% (*v*/*v*). All live-virus procedures were conducted in biosafety level 3 laboratories.

### 2.2. Construction of a Full-Length cDNA Clone

The full-length genome of the A/SKR/Yeoncheon/2017 virus was cloned by replacing the complete O1 Manisa genome in the pre-existing pBluescript II SK(+) vector. The viral genome was divided into two regions (small and large fragments), both of which were amplified via polymerase chain reaction (PCR). Viral RNA was extracted from the virus-infected BHK-21 culture supernatants using the QIAamp Viral RNA Mini Kit (Qiagen, Hilden, Germany), followed by elution in RNase-free water. Reverse transcription was performed using SuperScript IV Reverse Transcriptase (Invitrogen, Carlsbad, CA, USA) with an oligo(dT) primer annealed to the poly(A) tail at the 3′ end of the genome (42 °C, 60 min; inactivation, 70 °C, 5 min). The synthesized cDNA served as a PCR template. For the small fragment, PCR primers were designed to introduce NdeI and AvrII restriction sites at the 5′ and 3′ ends, respectively. The amplified region extended from the NdeI site upstream of the T7 promoter to the AvrII site downstream of the poly(C) tract. PCR was performed using Phusion High-Fidelity DNA Polymerase (Thermo Fisher Scientific, Waltham, MA, USA) in a 50 µL reaction under the following conditions: Initial denaturation at 98 °C for 30 s, 30 cycles of 98 °C for 10 s, 58 °C for 30 s, and 72 °C for 30 s/kb, and a final extension at 72 °C for 10 min. The PCR product was purified (Macherey-Nagel, Düren, Germany) and quantified. The plasmid backbone containing the O1 Manisa genome without the small fragment was isolated, ligated with the purified small fragment of the A/SKR/Yeoncheon/2017 virus, and incubated at 16 °C for 30 min with Ligation High ver. 2 (Toyobo, Osaka, Japan). The large genomic fragment of the A/SKR/Yeoncheon/2017 virus was amplified using primers containing AvrII (5′) and NotI (3′), in which the first adenine immediately following the poly(C) tract was substituted with thymine, and the fourth thymine was replaced with guanine for deliberate introduction of the AvrII restriction site, thereby facilitating efficient restriction enzyme-based cloning. Subsequently, PCR was performed under the following conditions: Initial denaturation at 98 °C for 30 s, 30 cycles of 98 °C for 10 s, 60 °C for 30 s, and 72 °C for 30 s/kb, and a final extension at 72 °C for 10 min. The PCR product (approximately 7.8 kb) was purified and inserted into a vector carrying the small fragment of the A/SKR/Yeoncheon/2017 virus. Then, O1 Manisa large region was excised via AvrII/NotI digestion, and the corresponding A/SKR/Yeoncheon/2017 fragment was ligated at a molar ratio of 1:5 using T4 DNA Ligase (New England Biolabs, Ipswich, MA, USA) at 16 °C for 30 min. All primer sequences used for cloning are listed in Table 1. Full-length genome sequencing was performed by Macrogen (Geumcheon-gu, Seoul, Republic of Korea)

### 2.3. Recovery of Recombinant FMDV

The recombinant plasmid was introduced into BHK/T7-9 cells stably expressing T7 RNA polymerase using Lipofectamine 3000 (Invitrogen, Waltham, MA, USA). Following transfection, the cells were incubated for three days at 37 °C in 5% CO_2_. The virus was recovered via freeze–thaw cycles and passaged in ZZ-R cells until cytopathic effects were observed. The rescued virus was sequentially propagated in adherent and suspended BHK-21 cells. Virus generation was confirmed via lateral-flow chromatographic immunoassay (VDRG FMDV 3Diff/Pan Ag Rapid kit; Median Diagnostics, Chuncheon, Korea) capable of detecting and differentiating the O, A, and Asia1 FMDV serotypes.

### 2.4. Transmission Electron Microscopy

The rescued virus particles were concentrated via polyethylene glycol precipitation and purified via ultracentrifugation using a 15–45% sucrose gradient (100,000× *g*, 4 h). The interface between the 30 and 35% layers was collected and recentrifuged under identical conditions, and the pellet was resuspended and dialyzed against 50 mM Tris buffer containing 300 mM KCl (pH 7.6) at 4 °C. The purified virus samples were applied onto Formvar-coated copper grids, stained with 1% uranyl acetate, and visualized using a transmission electron microscope (H-7100FA; Hitachi, Tokyo, Japan).

### 2.5. Virus Titration

Viral titers were determined via endpoint dilution on adherent BHK-21 monolayers and expressed as 50% tissue culture infectious doses (TCID_50_/mL), calculated using the Spearman–Kärber method [14].

### 2.6. Quantification of FMDV Particles

Virus-infected supernatants were mixed with chloroform (1:1 *v*/*v*; Merck KGaA, Darmstadt, Germany) and vigorously inverted for 5 min. After centrifugation (3000× *g*, 15 min, 4 °C), the aqueous phase was re-extracted with chloroform. The samples were centrifuged (16,000× *g*, 10 min), treated with benzonase (0.025 U/µL; Sigma-Aldrich) at 37 °C for 1 h, and filtered through the 0.22-µm Millex-GV Filter (Merck KGaA). Intact 146S particles were quantified via size-exclusion high-performance liquid chromatography using the TSKgel G4000PWXL (300 mm × 7.8 mm) and PWXL Guardcol (Tosoh Bioscience, Tokyo, Japan) columns on the Agilent 1260 Infinity II system. The mobile phase consisted of 30 mM Tris-HCl and 400 mM NaCl (pH 8.0) at 0.5 mL/min. Data were processed using the OpenLAB CDS ChemStation software (version 3.2.0.620), and 146S particle concentration (µg/mL) was determined as previously described [15].

### 2.7. Animal Experiment

A monovalent vaccine formulation was prepared with 15 µg of inactivated virus per dose, 1% saponin (Sigma-Aldrich), and 1% aluminum hydroxide gel (General Chemical, Mount Laurel, NJ, USA). Adjuvant ISA 206 VG (Seppic, Paris, France), pre-warmed to 30 °C, was incorporated at a 1:1 ratio to produce 2 mL per dose. The vaccine emulsion was maintained at 20 °C for 1 h in the dark and stored at 4 °C until use. Eight FMDV-seronegative pigs (two months old) were used; five received intramuscular immunization (2 mL/dose), whereas three served as unvaccinated controls. After vaccination at an external facility, the pigs were transported to a biosafety level 3 facility after three weeks and acclimated for one week before the experimental procedures. Throughout the study, the pigs were provided adequate feed and water. Blood samples were collected on 0, 7, 14, 21, and 28 d post-vaccination (dpv). On 28 dpv, all pigs were challenged via heel-bulb injection with 1 × 10^5^ TCID_50_ of O/Boeun/SKR/2017. Animals exhibiting clinical symptoms were immediately isolated to prevent secondary spread. Clinical scoring was performed as previously described [16]: Loss of appetite (1–2), lameness (1–2), coronary band pain (1–2), foot vesicles (up to 4), and oral lesions (up to 3); maximum score = 13. Protection was defined as the absence of visible lesions beyond the inoculation site within seven days post-challenge. Viral RNA in nasal/oral swabs and sera was quantified daily for one week after infection via real-time reverse transcription (RT)-PCR (AccuPower FMDV Real-Time RT-PCR Master Mix Kit; Bioneer, Daejeon, Republic of Korea).

### 2.8. Virus Neutralization Assay

Neutralizing antibody titers were measured according to the World Organization for Animal Health Terrestrial Manual guidelines [17]. The serum samples were heat-inactivated at 56 °C for 30 min and serially diluted two-fold from 1:2 in duplicate (50 µL/well). Each dilution was mixed with 100 TCID_50_ of virus and incubated at 37 °C for 1 h. Porcine kidney (LFBK) cells (0.5 × 10^6^ cells/mL; Plum Island Animal Disease Center, Orient, NY, USA) were added and cultured at 37 °C in 5% CO_2_ for 2–3 d. Virus neutralization titers were expressed as log_10_ reciprocal values of the highest serum dilution that completely neutralized 100 TCID_50_ of virus, calculated using the Spearman–Kärber method [14].

### 2.9. Enzyme-Linked Immunosorbent Assay

Type O FMDV-specific antibodies were quantified using the PrioCHECK FMDV Type A competition ELISA kit (Prionics, Lelystad, The Netherlands), following the manufacturer’s instructions. The serum samples were incubated on antigen-coated plates at 25 °C for 1 h, washed, and treated with horseradish peroxidase-conjugated monoclonal antibodies against FMDV structural proteins. Then, the optical density was measured at 450 nm after substrate development. The results were interpreted based on percentage inhibition, with values ≥ 50% considered positive and those <50% considered negative.

### 2.10. Statistical Analyses

All data are represented as the mean ± standard deviation of three independent experiments. Statistical significance was determined via one-way analysis of variance, followed by Tukey’s honest significant difference post hoc test (GraphPad Prism 8; GraphPad Software, San Diego, CA, USA). Different letters indicate the statistically significant differences (*p* < 0.05).

## 3. Results

### 3.1. Construction of an Infectious cDNA Clone

**Figure 1:**
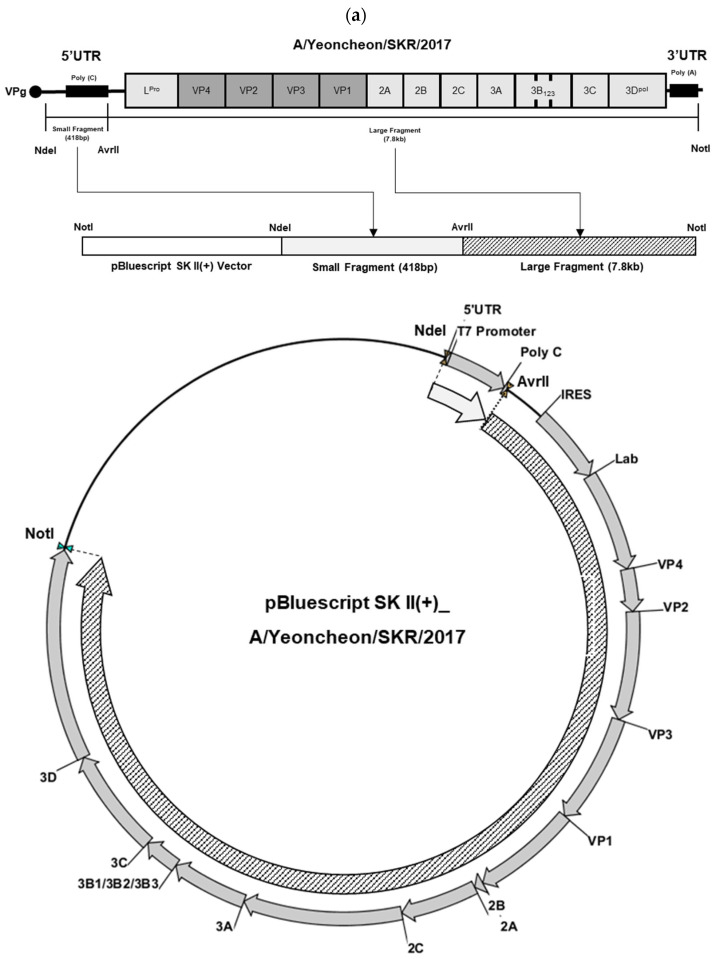

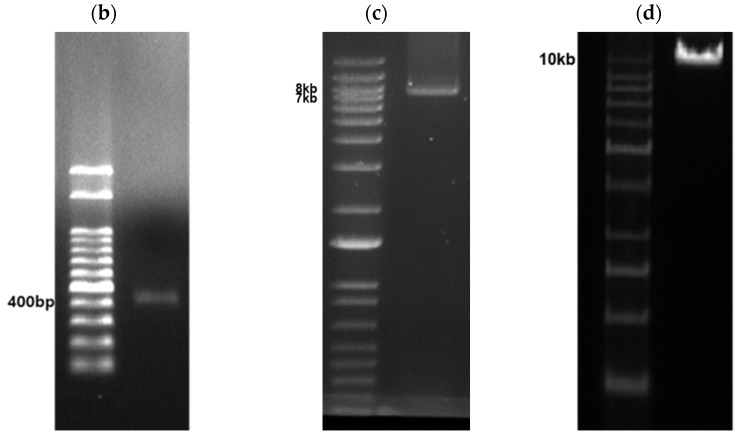
Construction of a full-length infectious cDNA clone of the A/SKR/Yeoncheon/2017 virus. (**a**) Schematic representation of the cloning strategy used for the construction of a full-length A/SKR/Yeoncheon/2017 cDNA clone. (**b**) Polymerase chain reaction (PCR) amplification of the small fragment of the A/SKR/Yeoncheon/2017 virus showing the expected 418 bp band on agarose gel. (**c**) PCR amplification of the large fragment (7.8 kb) of the A/SKR/Yeoncheon/2017 virus. (**d**) Full-length clone constructed by ligating the two fragments linearized via digestion with AvrII.

To generate an infectious clone corresponding to the domestic FMDV strain, the A/SKR/Yeoncheon/2017 viral genome was segmented into two regions, each of which was amplified via PCR (Figure 1a). The smaller genomic fragment (approximately 418 bp) derived from the A/SKR/Yeoncheon/2017 virus was successfully amplified and confirmed via agarose gel electrophoresis (Figure 1b). Subsequently, the larger genomic fragment (approximately 7.8 kb) of the A/SKR/Yeoncheon/2017 virus was amplified (Figure 1c). The infectious clone generated by ligating the large and small fragments was linearized with AvrII, producing a single band of approximately 11 kb (Figure 1d). Comparative sequence analysis with the parental A/SKR/Yeoncheon/2017 virus revealed that the amino acid at position 76 of VP3 was substituted from lysine to asparagine.

### 3.2. Rescue of Infectious FMDV from the cDNA Construct

**Figure 2:**
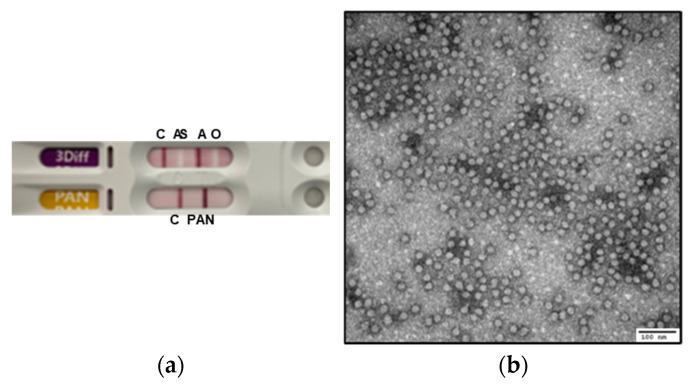
Confirmation of the serotype and morphology of the rescued A/SKR/Yeoncheon/2017 virus. (**a**) A rapid antigen test kit indicating that the virus is FMDV serotype A. (**b**) Transmission electron microscopy of the purified virus preparation revealing spherical particles with diameters of approximately 25–30 nm.

The recombinant plasmid was transfected into BHK/T7-9 cells, and the harvested supernatant was used to infect ZZ-R cells, which exerted cytopathic effects. The viral supernatant was initially propagated in adherent BHK-21 cells and subsequently expanded in suspension-adapted BHK-21 cells. Serotyping using a commercial diagnostic kit identified the rescued virus as FMDV serotype A (Figure 2a). The purified virus particles were visualized via transmission electron microscopy, revealing uniform 25–30 nm icosahedral virions (Figure 2b). These results confirmed the successful rescue of the infectious virus from the engineered full-length cDNA clone.

### 3.3. Antigen Productivity of the Rescued A/SKR/Yeoncheon/2017 Virus

**Figure 3:**
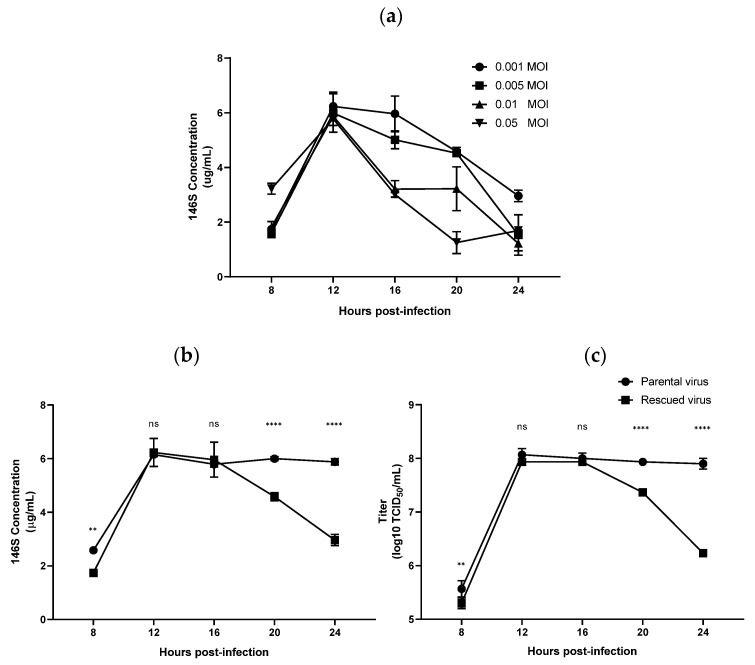
Antigen productivity and viral titer of the rescued A/SKR/Yeoncheon/2017 virus. (**a**) BHK-21 cells were infected with the rescued FMDV A/SKR/Yeoncheon/2017 virus at different multiplicities of infection, and 146S antigen productivity was quantified from 8 to 24 h post-infection (hpi). (**b**) 146S antigen productivity, and (**c**) the viral titers of the parental and rescued viruses were compared under the same virus infection conditions. Data are represented as the mean ± standard deviation of three independent experiments. Statistical comparisons were performed via unpaired Student’s *t*-test (ns, not significant; ** *p* < 0.01; **** *p* < 0.0001).

Antigen levels were analyzed according to viral concentration and infection time. As the viral inoculum decreased, antigen levels tended to increase over time. However, regardless of the initial viral concentration, the highest antigen levels were observed at 12 hpi (Figure 3a). Using this optimal multiplicity of infection of 0.001, viral titers and antigen concentrations were compared between the rescued and parental A/SKR/Yeoncheon/2017 viruses (Figure 3b,c). The parental virus exhibited the highest antigen level of 6.2 μg/mL and viral titer at 12 hpi, maintaining similar levels up to 24 h. The rescued virus showed the same amount of antigen (6.2 μg/mL) and viral titers at 12 hpi but demonstrated a sharp decline after 16 hpi. However, both viruses exhibited comparable antigen levels around 6.0 μg/mL at both 12 and 16 hpi, suggesting no significant difference in antigen productivity under optimal conditions.

### 3.4. Protective Efficacy of the Inactivated Vaccine Prepared from the Rescued Virus

**Figure 4:**
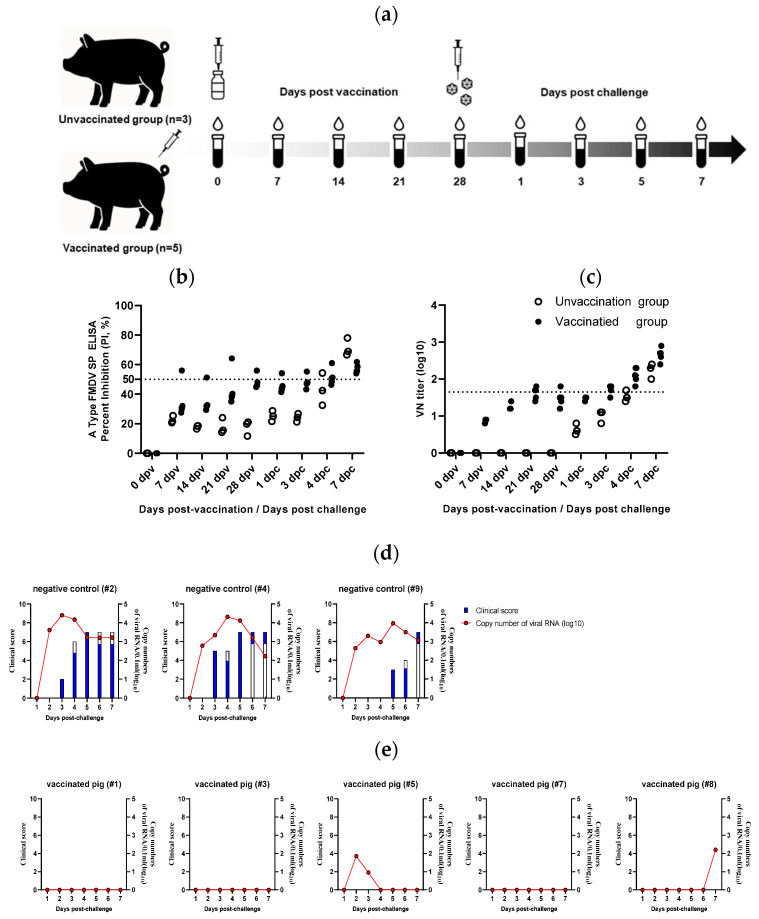
Protective efficacy of the rescued A/SKR/Yeoncheon/2017 virus in pigs. Pigs were immunized with an inactivated vaccine derived from the rescued A/SKR/Yeoncheon/2017 virus and subsequently challenged with a homologous field strain to evaluate the protective immunity. (**a**) Diagram showing the vaccination and challenge timeline, including serum sampling points. (**b**) Structural protein enzyme-linked immunosorbent assay (ELISA) results for the serum samples collected weekly after vaccination and challenge. The dotted line indicates the threshold for a positive result. (**c**) Virus neutralization titers in the same serum samples. The dotted line indicates a titer of ≥1.65 log 10. Clinical symptoms and FMDV RNA levels in the nasal/oral swab samples of (**d**) unvaccinated and (**e**) vaccinated pigs after virus challenge.

To determine the protective capacity of the rescued A/SKR/Yeoncheon/2017 virus, it was inactivated and purified via sucrose density gradient ultracentrifugation. A monovalent test vaccine containing 15 µg of antigen per dose was formulated and intramuscularly administered to pigs. Serum and nasal/oral swab samples were collected weekly to assess antibody development (Figure 4a). Enzyme-linked immunosorbent assay revealed that all vaccinated pigs exhibited antibody levels near the cutoff of 50% percentage inhibition (45.5–56.0%) used to determine seropositivity at 28 dpv, immediately before virus challenge (Figure 4b). In the virus neutralization tests, all vaccinated pigs showed titers of 1.2–1.8 log_10_ (Figure 4c). Upon challenge with the virulent A/SKR/Yeoncheon/2017 virus, all non-vaccinated control pigs exhibited clear clinical signs of FMD, including vesicular lesions and lameness, and viral RNA was readily detected in the nasal/oral secretions (Figure 4d). In contrast, none of the vaccinated pigs exhibited any clinical symptoms (Figure 4e), and two of five pigs showed viral RNA in their nasal/oral secretions. Overall, the inactivated vaccine derived from the rescued A/SKR/Yeoncheon/2017 virus effectively protected against the homologous FMDV challenge in pigs.

## 4. Discussion

In this study, we successfully constructed and characterized the first infectious cDNA clone derived from a field-isolated serotype A FMDV strain in Korea, A/SKR/Yeoncheon/2017, a virus previously identified for its superior antigen productivity following adaptation to suspension culture.

The full-length viral genome was cloned into two large fragments using two restriction enzymes. Previous studies have constructed infectious clones of FMDV using multiple smaller fragments [18,19,20,21,22]. Moreover, recent studies have described simplified approaches with overlap-based recombination methods, such as Gibson assembly, which eliminate the need for restriction enzyme digestion [23].

Unlike other viral vaccines, the potency of FMD vaccine antigens is measured by the concentration of intact 146S particles, not the amount of individual viral proteins [24,25]. This distinction indicates that the immunogenicity of the assembled virion differs significantly from that of individual structural proteins. Indeed, thermal degradation of FMDV into 12S pentamers markedly reduces vaccine efficacy [26,27].

Interestingly, viral titer and antigen yield are not always proportional; viruses with similar titers can produce different antigen levels [13,28]. In this study, the virus rescued from the infectious clone exhibited an antigen yield comparable to that of the parental virus under optimal conditions. However, antigen levels decreased after 16 hpi, possibly due to the amino acid substitution from lysine to asparagine at position 76 of the VP3 protein during cloning. FMDV shows diverse mutations during its cell adaptation phase, with the specific sites varying across viral strains rather than following a general rule. However, the VP3 position identified here has not been commonly implicated in adaptation-associated mutational hotspots [10]. This underscores the need for further experiments—such as targeted mutagenesis or structural modeling—to clarify its functional significance.

Notably, this study achieved higher antigen production (>6 μg/mL) than previous studies [29,30,31]. Previous studies have reported antigen levels of approximately 1 µg/mL in viral infection supernatants [32,33,34]. High antigen productivity is a decisive advantage for industrial-scale vaccine manufacturing, directly influencing production cost. Additional comparative analyses across diverse serotype A strains may reveal the molecular features that confer this superior yield and help inform the design of next-generation vaccine backbones.

Pigs vaccinated with the inactivated rescued virus (15 μg/dose) exhibited antibody titers around the cutoff value (1/45) and were completely protected upon virus challenge. A protection rate of approximately 75% is achieved at antibody titers of 1.26–1.64 log_10_ [35]. This finding aligns with previous evidence demonstrating that certain type A vaccine strains can confer strong protection even at antibody titers below conventional thresholds [36]. We believe that partial humoral immunity combined with cell-mediated immune responses contributed to protection.

Among the seven FMDV serotypes, O and SAT types exhibit relatively low physical stability, and several studies have attempted to enhance their stability by substituting specific genomic regions [5,37,38]. Finally, the infectious clone developed in this study offers several practical advantages. By serving as a genetically stable, high-yield backbone, it enables rapid replacement of the capsid-encoding region with that of emerging field strains—eliminating the need for lengthy adaptation procedures. Additionally, it provides a platform for engineering viruses with enhanced physicochemical stability, which is especially relevant for serotypes such as O and SAT that naturally exhibit lower capsid robustness. Ultimately, this technology supports a faster, more flexible response to FMD incursions and contributes to the long-term goal of establishing full domestic vaccine self-sufficiency.

## 5. Conclusions

To the best of our knowledge, this study reports the first successful construction of an infectious cDNA clone derived from a field-isolated serotype A FMDV strain in South Korea, A/SKR/Yeoncheon/2017. Moreover, the established A/SKR/Yeoncheon/2017 virus backbone will serve as a versatile platform to develop high-yield FMD vaccine candidates via epitope substitution in various type A viruses that may enter the country in the future.

## Figures and Tables

**Table 1:** Primer sequences used to amplify the small and large fragments used for the construction of the A/SKR/Yeoncheon/2017 infectious clone.

Region	Primer	Sequence (5′→3′)
Small fragment	Forward	GAC*CATATG*TAATACGACTCACTATAGGGTTGAAAGGGGGCGTTAGGGT
	Reverse	TTG*CCTAGG*GGGGGGGGGGGGGGGGGGAAAGGTGGGCTTCGGGT
Large fragment	Forward	TTG*CCTAGG*ACTACCGTCGTTCCCGACGT
	Reverse	GAC*GCGGCCGC*TCTAGAACTAGTTTTTTTTTTTTTTTTTTTGGAAGAGGAAGCGGGAA

The T7 promoter sequence is underlined, and the restriction enzyme recognition sites are indicated in italics.

## Data Availability

The original contributions presented in this study are included in the article. Further inquiries can be directed to the corresponding author.

## References

[1] Mielke S.R., Lendzele S., Delgado A.H., Abdoulmoumini M., Dickmu S., Garabed R. (2023). Patterns of foot-and-mouth disease virus detection in environmental samples in an endemic setting. Front. Vet. Sci..

[2] Kim S.W., Lee S.H., Kim H.-H., Shin S.-H., Park S.-H., Park J.-H., Kim J., Park C.-K. (2024). Evaluation of swine protection with three commercial foot-and-mouth disease vaccines against heterologous challenge with type A ASIA/G-VII lineage viruses. Vaccines.

[3] Curry S., Fry E., Blakemore W., Abu-Ghazaleh R., Jackson T., King A., Lea S., Newman J., Stuart D. (1997). Dissecting the roles of VP0 cleavage and RNA packaging in picornavirus capsid stabilization: The structure of empty capsids of foot-and-mouth disease virus. J. Virol..

[4] Li C., Shi J., Wang H., Rivera-Serrano E.E., Yang D., Zhou G., Sun C., Cameron C.E., Yu L. (2020). Polymerase fidelity contributes to foot-and-mouth disease virus pathogenicity and transmissibility in vivo. J. Virol..

[5] Kotecha A., Seago J., Scott K., Burman A., Loureiro S., Ren J., Porta C., Ginn H.M., Jackson T., Perez-Martin E. (2015). Structure-based energetics of protein interfaces guides foot-and-mouth disease virus vaccine design. Nat. Struct. Mol. Biol..

[6] Bai X.-W., Bao H.-F., Li P.-H., Ma X.-Q., Sun P., Bai Q.-F., Zhang M., Yuan H., Chen D.-D., Li K. (2019). Engineering responses to amino acid substitutions in the VP0-and VP3-coding regions of PanAsia-1 strains of foot-and-mouth disease virus serotype O. J. Virol..

[7] Seeyo K.B., Nishi T., Kawaguchi R., Ungvanijban S., Udon R., Fukai K., Yamakawa M., Rukkwamsuk T. (2020). Evolution of antigenic and genetic characteristics of foot-and-mouth disease virus serotype A circulating in Thailand, 2007–2019. Virus Res..

[8] Park M.-Y., Han Y.J., Choi E.-J., Kim H., Pervin R., Shin W., Kwon D., Kim J.M., Pyo H.M. (2021). Post-vaccination monitoring to assess foot-and-mouth disease immunity at population level in Korea. Front. Vet. Sci..

[9] Hwang J.-H., Lee G., Kim A., Park J.-H., Lee M.J., Kim B., Kim S.-M. (2021). A vaccine strain of the A/ASIA/Sea-97 lineage of foot-and-mouth disease virus with a single amino acid substitution in the P1 region that is adapted to suspension culture provides high immunogenicity. Vaccines.

[10] Dill V., Eschbaumer M. (2020). Cell culture propagation of foot-and-mouth disease virus: Adaptive amino acid substitutions in structural proteins and their functional implications. Virus Genes.

[11] Lee S.-Y., Lee Y.-J., Kim R.-H., Park J.-N., Park M.-E., Ko M.-K., Choi J.-H., Chu J.-Q., Lee K.-N., Kim S.-M. (2017). Rapid engineering of foot-and-mouth disease vaccine and challenge viruses. J. Virol..

[12] Medina G.N., Spinard E., Azzinaro P.A., Rodriguez-Calzada M., Gutkoska J., Kloc A., Rieder E.A., Taillon B.E., Mueller S., de Los Santos T. (2023). Deoptimization of FMDV P1 region results in robust serotype-independent viral attenuation. Viruses.

[13] Park S.-H., Lee S.-Y., Kim J.-S., Kim A.-Y., Park S.-Y., Lee J.-H., Lee M., Kim H., Lee S.-I., Kang N.-Y. (2021). Scale-up production of type O and a foot-and-mouth disease bivalent vaccine and its protective efficacy in pigs. Vaccines.

[14] Kärber G. (1931). Beitrag zur kollektiven Behandlung pharmakologischer Reihenversuche. Naunyn-Schmiedebergs Arch. Exp. Pathol. Pharmakol..

[15] Spitteler M.A., Romo A., Magi N., Seo M.-G., Yun S.-J., Barroumeres F., Régulier E.G., Bellinzoni R. (2019). Validation of a high performance liquid chromatography method for quantitation of foot-and-mouth disease virus antigen in vaccines and vaccine manufacturing. Vaccine.

[16] Alves M., Guzylack-Piriou L., Juillard V., Audonnet J.-C., Doel T., Dawson H., Golde W., Gerber H., Peduto N., McCullough K. (2009). Innate immune defenses induced by CpG do not promote vaccine-induced protection against foot-and-mouth disease virus in pigs. Clin. Vaccine Immunol..

[17] WOAH (2022). WOAH Terrestrial Manual 2022. Foot and Mouth Disease (Infection with Foot and Motu Disease Virus).

[18] Liu G., Liu Z., Xie Q., Chen Y., Bao H., Chang H., Liu X. (2004). Generation of an infectious cDNA clone of an FMDV strain isolated from swine. Virus Res..

[19] Xin A., Li H., Li L., Liao D., Yang Y., Zhang N., Chen B. (2009). Genome analysis and development of infectious cDNA clone of a virulence-attenuated strain of foot-and-mouth disease virus type Asia 1 from China. Vet. Microbiol..

[20] Zibert A., Maass G., Strebel K., Falk M., Beck E. (1990). Infectious foot-and-mouth disease virus derived from a cloned full-length cDNA. J. Virol..

[21] Lian K., Yang F., Zhu Z., Cao W., Jin Y., Li D., Zhang K., Guo J., Zheng H., Liu X. (2015). Recovery of infectious type Asia1 foot-and-mouth disease virus from suckling mice directly inoculated with an RNA polymerase I/II-driven unidirectional transcription plasmid. Virus Res..

[22] Chang Y., Zheng H., Shang Y., Jin Y., Wang G., Shen X., Liu X. (2009). Recovery of infectious foot-and-mouth disease virus from full-length genomic cDNA clones using an RNA polymerase I system. Acta Biochim. Biophys. Sin..

[23] Semkum P., Thangthamniyom N., Chankeeree P., Keawborisuth C., Theerawatanasirikul S., Lekcharoensuk P. (2023). The application of the Gibson assembly method in the production of two pKLS3 vector-derived infectious clones of foot-and-mouth disease virus. Vaccines.

[24] Doel T., Collen T. (1982). Qualitative assessment of 146 S particles of foot-and-mouth disease virus in preparations destined for vaccines. J. Biol. Stand..

[25] Harmsen M.M., Seago J., Perez E., Charleston B., Eblé P.L., Dekker A. (2017). Isolation of single-domain antibody fragments that preferentially detect intact (146S) particles of foot-and-mouth disease virus for use in vaccine quality control. Front. Immunol..

[26] Li H., Liu P., Dong H., Dekker A., Harmsen M.M., Guo H., Wang X., Sun S. (2024). Foot-and-mouth disease virus antigenic landscape and reduced immunogenicity elucidated in atomic detail. Nat. Commun..

[27] Doel T., Chong W. (1982). Comparative immunogenicity of 146S, 75S and 12S particles of foot-and-mouth disease virus. Arch. Virol..

[28] Dill V., Zimmer A., Beer M., Eschbaumer M. (2020). Targeted modification of the foot-and-mouth disease virus genome for quick cell culture adaptation. Vaccines.

[29] Barteling S., Meloen R. (1974). A simple method for the quantification of 140 S particles of foot-and-mouth disease virus (FMDV). Arch. Gesamte Virusforsch..

[30] Van Rensburg H.G., Mason P.W. (2002). Construction and evaluation of a recombinant foot-and-mouth disease virus: Implications for inactivated vaccine production. Ann. N. Y. Acad. Sci..

[31] Li X.-R., Yang Y.-K., Wang R.-B., An F.-L., Zhang Y.-D., Nie J.-Q., Ahamada H., Liu X.-X., Liu C.-L., Deng Y. (2019). A scale-down model of 4000-L cell culture process for inactivated foot-and-mouth disease vaccine production. Vaccine.

[32] Wu P., Rodríguez Y.Y., Hershey B.J., Tadassa Y., Dodd K.A., Jia W. (2021). Validation of a binary ethylenimine (BEI) inactivation procedure for biosafety treatment of foot-and-mouth disease viruses (FMDV), vesicular stomatitis viruses (VSV), and swine vesicular disease virus (SVDV). Vet. Microbiol..

[33] Aarthi D., Rao K.A., Robinson R., Srinivasan V. (2004). Validation of binary ethyleneimine (BEI) used as an inactivant for foot and mouth disease tissue culture vaccine. Biologicals.

[34] Rweyemamu M., Umehara O., Giorgi W., Medeiros R., Lucca Neto D., Baltazar M. (1989). Effect of formaldehyde and binary ethyleneimine (BEI) on the integrity of foot and mouth disease virus capsid. Rev. Sci. Tech..

[35] Kim J., Lee S.-H., Kim H.-H., Shin S.-H., Park S.-H., Park J.-H., Park C.-K. (2024). An alternative serological measure for assessing foot-and-mouth disease vaccine efficacy against homologous and heterologous viral challenges in pigs. Vaccines.

[36] Hwang J.-H., Lee K.-N., Kim S.-M., Kim H., Park S.-H., Kim D.-W., Cho G., Lee Y.-H., Lee J.-S., Park J.-H. (2024). Enhanced effects of ISA 207 adjuvant via intradermal route in foot-and-mouth disease vaccine for pigs. Vaccines.

[37] Yuan H., Li P., Bao H., Sun P., Bai X., Bai Q., Li N., Ma X., Cao Y., Fu Y. (2020). Engineering viable foot-and-mouth disease viruses with increased acid stability facilitate the development of improved vaccines. Appl. Microbiol. Biotechnol..

[38] Scott K.A., Kotecha A., Seago J., Ren J., Fry E.E., Stuart D.I., Charleston B., Maree F.F. (2017). SAT2 foot-and-mouth disease virus structurally modified for increased thermostability. J. Virol..

